# Silver staining (Campbell-Switzer) of neuronal α-synuclein assemblies induced by multiple system atrophy and Parkinson’s disease brain extracts in transgenic mice

**DOI:** 10.1186/s40478-019-0804-5

**Published:** 2019-09-16

**Authors:** Isabelle Lavenir, Daniela Passarella, Masami Masuda-Suzukake, Annabelle Curry, Janice L. Holton, Bernardino Ghetti, Michel Goedert

**Affiliations:** 1 MRC Laboratory of Molecular Biology, Cambridge, CB2 0QH UK; 2 Queen Square Institute of Neurology, University College, London, WC1N 1PJ UK; 3 Department of Pathology and Laboratory Medicine, Indiana University School of Medicine, Indianapolis, IN 46202 USA

**Keywords:** α-Synuclein, Multiple system atrophy, Parkinson’s disease, Seeding, Silver staining, Transgenic

## Abstract

Synucleinopathies [Parkinson’s disease (PD), dementia with Lewy bodies (DLB) and multiple system atrophy (MSA)] share filamentous α-synuclein assemblies in nerve cells and glial cells. We compared the abilities of brain extracts from MSA and PD patients to induce neuronal α-synuclein assembly and neurodegeneration following intracerebral injection in heterozygous mice transgenic for human mutant A53T α-synuclein. MSA extracts were more potent than PD extracts in inducing α-synuclein assembly and in causing neurodegeneration. MSA assemblies were Campbell-Switzer- and Gallyas-silver-positive, whereas PD assemblies were only Campbell-Switzer-positive, in confirmation of previous findings. However, induced α-synuclein inclusions were invariably Campbell-Switzer-positive and Gallyas-negative, irrespective of whether MSA or PD brain extracts were injected. The α-synuclein inclusions of non-injected homozygous mice transgenic for A53T α-synuclein were also Campbell-Switzer-positive and Gallyas-negative. These findings demonstrate that transgene expression and its intracellular environment dominated over the silver staining properties of the conformers of assembled α-synuclein.

## Introduction

The ordered assembly of α-synuclein into abnormal filaments defines a group of neurodegenerative diseases called synucleinopathies [18]. α-Synuclein was linked to Parkinson’s disease (PD), when a dominantly inherited missense mutation (A53T) in *SNCA*, the α-synuclein gene, was found to cause a familial form of PD [39]. Subsequently, genome-wide association studies also identified α-synuclein as a significant risk factor for idiopathic PD [32]. α-Synuclein is the major component of Lewy bodies and Lewy neurites, the intraneuronal filamentous assemblies found in all patients with PD, with or without dementia, and in patients with dementia with Lewy bodies (DLB) [44, 45]. α-Synuclein not only accumulates in Lewy pathology, but it can also template its assembly. Injection of misfolded α-synuclein induces assembly of endogenous protein into phosphorylated assemblies that resemble Lewy bodies [26, 31]. Neurons bearing Lewy bodies eventually die [19, 33].

Filamentous inclusions of multiple system atrophy (MSA) are also made of α-synuclein [43, 47, 51]. MSA is more aggressive than PD and DLB, with an interval between diagnosis and death of approximately 9 years [11]. The defining lesion of MSA is the presence of α-synuclein inclusions in oligodendrocytes, the majority of which are in the form of cytoplasmic inclusions [glial cytoplasmic inclusions (GCIs) or Papp-Lantos inclusions [34, 35]]. Smaller numbers of filamentous α-synuclein inclusions are also present in the nuclei, cytoplasm and processes of some neurons [8]. Inclusions comprise α-synuclein phosphorylated at S129 [1, 13].

Much previous work on seeded aggregation used M83 mice, which are transgenic for human mutant A53T α-synuclein, under the control of the prion protein promoter [16]. Homozygous mice (M83^+/+^) develop abundant neuronal inclusions made of filamentous pS129 α-synuclein and neurodegeneration when aged 8–16 months, whereas heterozygotes (M83^+/−^) show no α-synuclein inclusions or neurodegeneration until 20 months of age. Inclusions are most abundant throughout spinal cord and hindbrain, followed by midbrain, thalamus and hypothalamus.

Experimental studies established that the intracellular milieu of oligodendrocytes is essential for the formation of GCI-specific conformers of assembled α-synuclein [33]. Previous investigations using mouse models of seeded pathology through intracerebral injections with brain samples from patients with either PD or MSA showed that MSA extracts were capable of inducing neuronal α-synuclein pathology in transgenic mice [40, 52]. Thus, homogenates from the brains of patients with MSA induced the formation of abundant α-synuclein inclusions and neurodegeneration characterized by motor symptoms in M83^+/−^ mice. In contrast, injection of brain homogenates from the brains of PD patients failed to induce the formation of either α-synuclein inclusions or neurodegeneration. These findings lent support to the view that different conformers of assembled α-synuclein are typical of PD and MSA.

Although the filamentous inclusions of MSA and PD are made of modified, assembled α-synuclein, they can be distinguished using silver staining, consistent with the presence of distinct conformers [35, 49]. Thus, GCIs and neuronal inclusions exhibit argyrophilia with both Campbell-Switzer and Gallyas, whereas Lewy pathology stains with Campbell-Switzer, but not Gallyas.

We injected brain homogenates from 7 MSA and 5 PD cases, all neuropathologically confirmed, into the hippocampus and overlying cerebral cortex of M83^+/−^ mice. Thirty mice injected with MSA brain extracts developed abundant neuronal α-synuclein inclusions and neurodegeneration, with an incubation time of 167 ± 17 days. The same was true of 7 of 17 mice injected with PD extracts (incubation time of 286 ± 62 days). By 18 months of age, 5 additional mice had developed α-synuclein inclusions, in the absence of neurodegeneration.

Inclusions were positive with Campbell-Switzer and negative with Gallyas silver, irrespective of whether MSA or PD extracts were injected. The same was true of assembled recombinant α-synuclein, which induced the formation of abundant α-synuclein inclusions and caused neurodegeneration (incubation time of 145 ± 10 days). α-Synuclein inclusions of non-injected M83^+/+^ mice were also Campbell-Switzer-positive and Gallyas-negative.

## Materials and methods

### Transgenic mice

The M83 transgenic mouse line, which expresses human mutant A53T α-synuclein under the control of the mouse prion protein promoter [16], was purchased from the Jackson Laboratory (stock number 004479). Mice heterozygous and homozygous for the transgene were used. All experiments were carried out in compliance with the Animals (Scientifc Procedures) Act of 1986 and were approved by the local Animal Welfare and Ethical Review Board.

### Human brain tissues

Frozen brain tissues from neuropathologically confirmed cases of MSA and PD were obtained from the Queen Square Brain Bank for Neurological Disorders (London, UK) and the tissue collection at Indiana University (Indianapolis, USA). Tissues were homogenised in phosphate-buffered saline (PBS) (200 mg/ml), sonicated (Misonix: output 2 for 5 × 0.9 s) and centrifuged in the cold at 3000 g for 5 min. Supernatants were aliquoted, snap frozen and stored at − 80 °C until use.

### Recombinant human α-synuclein

Full-length human α-synuclein was expressed and purified, as described [23, 53]. It was assembled into filaments by incubating 400 μM at 37 °C for 48 h with constant agitation at 450 rpm.

### Intracerebral injection

Three-month-old heterozygous M83 mice were anaesthetised with isoflurane and injected unilaterally with 5 μl human brain extract or 5 μl assembled recombinant human α-synuclein (400 μM), as described for tau assemblies [6]; 2.5 μl were injected into the right hippocampus (A/P, − 2.5 mm; M/L, + 2.0 mm; D/V, − 2.0 mm) and 2.5μl into the overlying cerebral cortex (A/P, − 2.5 mm; M/L, + 2.0 mm; D/V, − 1.0 mm) at a speed of 1.25 μl/min. Following injection, the needle was kept in place for another 3 min. Mice were given analgesia (Rimadyl, 4 mg/kg) prior to surgery and were placed on a heat mat; their body temperatures were monitored throughout surgery and they were placed in a heat cabinet after surgery to aid recovery.

### Survival

Following stereotaxic brain injections, mice were monitored weekly for signs of motor impairment. When they reached hind limb paralysis, they were humanely killed and their brains and spinal cords collected. Kaplan-Meier survival curves were produced using Graphpad Prism 7.

### Dot blotting

The levels of α-synuclein phosphorylated at S129 were determined by dot blotting (Minifold I Spot-Blot System, GE Healthcare), using human brain samples diluted 1:1000, and run in quadruplicate. Dried nitrocellulose membranes (Amersham) were blocked, incubated with a polyclonal pS129 α-synuclein antibody (ab18467, Abcam, 1:5000) for 3 h at room temperature, followed by secondary antibody (1:4000) for 1 h. Chemiluminescence and ImageJ were used to quantify the signal. Phosphorylation of purified recombinant human α-synuclein at S129 using casein kinase-2 (New England Biolabs) was done as described [42]. Serial dilutions (0.5–3.5 ng) were used as standard. The linear parts of the standard curves were used to measure the concentrations of pS129 α-synuclein.

### Immunohistochemistry and silver staining

Mice were terminally anaesthetised and transcardially perfused with 20 ml cold PBS, followed by 20 ml 4% paraformaldehyde in 0.1 M phosphate buffer. Brains and spinal cords were dissected and postfixed overnight. Fixed tissues were paraffin-embedded and 8 μm sections cut. Following deparaffinisation, the sections were incubated in blocking buffer [PBS + 0.1% Triton X-100 (PBST) + 10% foetal calf serum] for 15 min at room temperature, followed by an overnight incubation with primary antibody specific for pS129 α-synuclein (ab51253, Abcam, 1:5000 dilution) in blocking buffer. After three rinses with PBST, the sections were incubated with biotin-conjugated secondary anti-rabbit antibody (1:200 dilution) for 1 h at room temperature. The antigen was visualised with the Vector VIP substrate kit (Vector Laboratories). Fixed, deparaffinised tissue sections were stained using Campbell-Switzer [4, 5] or Gallyas [6, 15] silver, as described. All sections were counterstained with haematoxylin and coverslipped using Pertex mounting medium.

## Results

We homogenised and sonicated cerebellum (7 cases of MSA) and substantia nigra (5 cases of PD) from neuropathologically confirmed cases of disease (200 mg/ml) and injected 2.5 μl into the hippocampus and 2.5 μl into the overlying cerebral cortex of heterozygous M83 mice transgenic for human mutant A53T α-synuclein. Recombinant assembled human α-synuclein was used as a positive and cerebellar extract from a neurologically normal individual as a negative control. Upon development of hindlimb paralysis, the mice were culled, their brains and spinal cords dissected and stained for pS129 α-synuclein, as well as Campbell-Switzer and Gallyas silver. Mice without hindlimb paralysis were culled at 18 months of age.

**Fig. 1:**
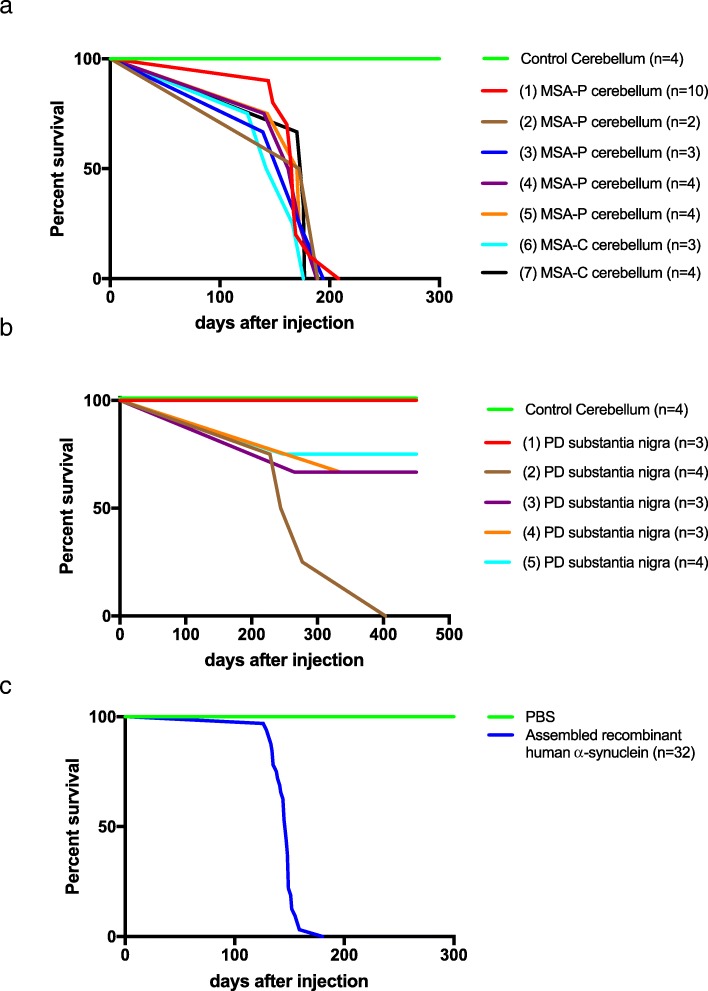
Survival of heterozygous mice transgenic for human mutant A53T α-synuclein following intracerebral injection of (**a**) cerebellar extracts from seven patients with multiple system atrophy (MSA) and (**b**) substantia nigra extracts from five patients with Parkinson’s disease (PD). Cerebellum from a neurologically unaffected individual served as control. Five MSA cases were of the parkinsonian type (MSA-P) and two cases of the cerebellar type (MSA-C). Assembled recombinant human α-synuclein was also injected (**c**)

**Fig. 2:**
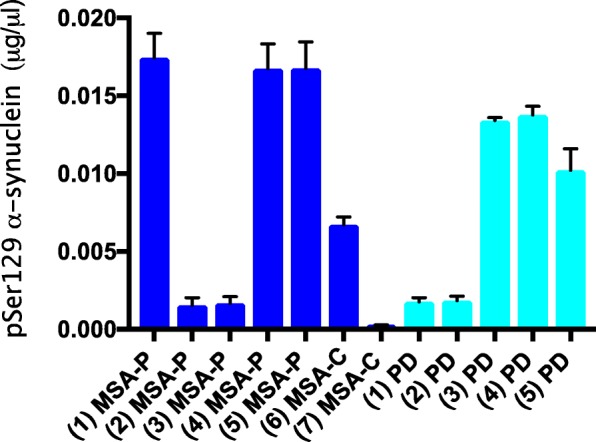
Dot blotting was used to measure the levels of pS129 α-synuclein in cerebellar extracts from the seven cases of multiple system atrophy (MSA) and substantia nigra extracts from the five cases of Parkinson’s disease (PD) that were used for intracerebral injection. Recombinant α-synuclein phosphorylated at S129 was used for the standard curves

Thirty heterozygous M83 mice were injected with cerebellar extract from 7 cases of MSA. All injected mice developed hindlimb paralysis with an average incubation time of 167 ± 17 days (Table 1; Fig. 1). Assembled α-synuclein was detected in the central nervous system (Figs. 4 and 5), with amounts and distributions similar to those previously described for homozygous M83 mice [16]. We injected 17 heterozygous M83 mice with substantia nigra extract from 5 cases of PD. Only 7 mice developed hindlimb paralysis, with an average incubation time of 286 ± 62 days (Table 2; Fig. 1). Assembled α-synuclein was detected in the central nervous system, with a distribution similar to that seen in homozygous mice (Figs. 4 and 5). However, the number of inclusions was approximately two-fold less. The remaining 10 mice were culled at 18 months of age. Five mice showed staining for α-synuclein phosphorylated at S129, with a similar distribution of pathology to that described in homozygous M83 mice, but significantly fewer inclusions in a given region. As a positive control, 32 heterozygous M83 mice were injected with 30 μg assembled recombinant human α-synuclein. All mice developed hindlimb paralysis and extensive pS129-α-synuclein immunoreactivity, with an average incubation time of 145 ± 10 days (Figs. 1, 4 and 5). As a negative control, cerebellar extract from a 64 year old neurologically normal individual was injected into 4 heterozygous M83 mice. At 18 months of age, there were no motor symptoms or staining for pS129 α-synuclein. Dot blotting showed that the levels of α-synuclein phosphorylated at S129 varied between cases (Fig. 2). Thus, approximately 1 ng assembled α-synuclein from MSA case 7 had a similar effect as approximately 85 ng from MSA case 1. However, this was unlikely to account for the differences between MSA and PD. The levels of assembled α-synuclein of MSA cases 2 and 3 were similar to those of PD cases 1 and 2.

**Table 1:** Motor impairment of heterozygous mice transgenic for human mutant A53T α-synuclein following intracerebral injection of brain extracts from multiple system atrophy (MSA)

Human brain extract	Age at death	Mice injected	Mice with motor impairment	Survival (days)	SD
(1) MSA-P	68	10	10	168	21
(2) MSA-P	75	2	2	181	12
(3) MSA-P	82	3	3	166	28
(4) MSA-P	65	4	4	166	20
(5) MSA-P	83	4	4	168	19
(6) MSA-C	69	3	3	174	4
(7) MSA-C	60	4	4	152	23

**Table 2:** Motor impairment of heterozygous mice transgenic for human mutant A53T α-synuclein following intracerebral injection of brain extracts from Parkinson’s disease (PD)

Human brain extract	Age at death	Mice injected	Mice with motor impairment	Survival (days)	SD	Survivors to 18 Mo	Survivors with assembled α-synclein
(1) PD	76	3	0	–	–	3	2
(2) PD	83	4	4	288	79	–	–
(3) PD	92	3	1	265	–	2	1
(4) PD	68	3	1	335	–	2	1
(5) PD	74	4	1	–	–	3	1

**Fig. 3:**
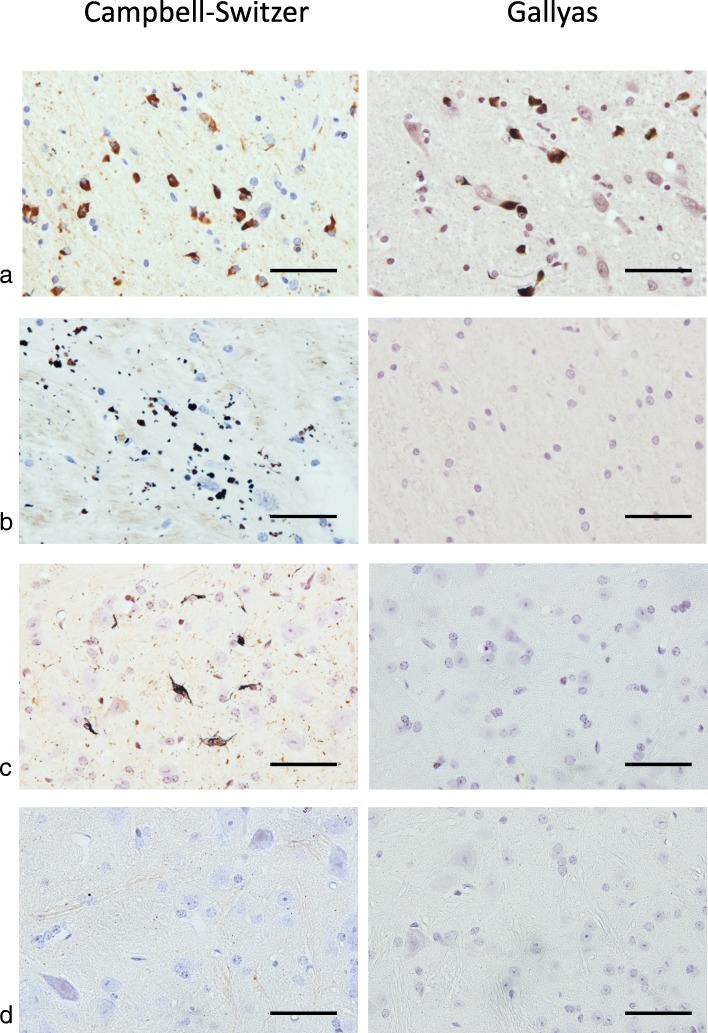
Silver staining (Campbell-Switzer and Gallyas) of tissue sections from (**a**) brainstem of multiple system atrophy (MSA-P) case 4, (**b**) substantia nigra of Parkinson’s disease (PD) case 5 and lumbar spinal cord of non-injected (**c**) homozygous (tgM83^+/+^) and (**d**) heterozygous (tgM83^+/−^) mice transgenic for human mutant A53T α-synuclein. Only MSA sections were Gallyas-positive. They were also stained by Campbell-Switzer, as were PD and tgM83^+/+^ sections. TgM83^+/−^ sections were silver-negative. Scale bars, 50 μm

α-Synuclein pathologies of PD and MSA can be distinguished by silver staining [49]. PD pathology is stained by Campbell-Switzer, but not Gallyas silver. By contrast, MSA pathology is stained by both Campbell-Switzer and Gallyas silver. We confirmed these findings (Table 3; Fig. 3). Cases of PD were positive for Campbell-Switzer, but not Gallyas, whereas all cases of MSA were positive for both silver stains. Like PD, brain and spinal cord from homozygous M83 mice were positive for Campbell-Switzer, but negative for Gallyas silver (Table 3; Fig. 3). Tissue sections from heterozygous M83 mice were silver-negative.

**Table 3:** Silver staining of brain sections from multiple system atrophy (MSA) and Parkinson’s disease (PD) patients. Cerebellum was used for MSA cases 2, 3, 6, 7, brainstem for cases 4, 5 and basal ganglia for case 1. Substantia nigra was used for PD cases 1, 3, 4, 5 and cingulate cortex for case 2

Human tissue	Gallyas silver	Campbell-Switzer silver
(1) MSA-P	+	+
(2) MSA-P	+	+
(3) MSA-P	+	+
(4) MSA-P	+	+
(5) MSA-P	+	+
(6) MSA-C	+	+
(7) MSA-C	+	+
(1) PD	–	+
(2) PD	–	+
(3) PD	–	+
(4) PD	–	+
(5) PD	–	+

**Fig. 4:**
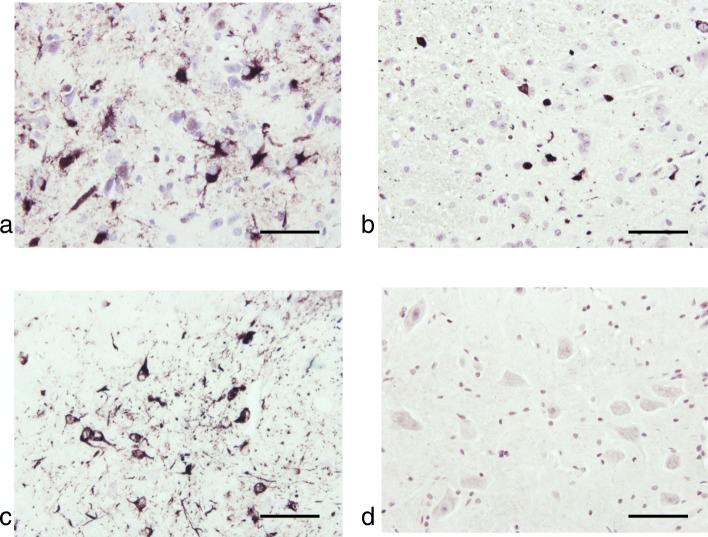
Immunohistochemistry for pS129-α-synuclein of lumbar spinal cord from heterozygous mice transgenic for human mutant A53T α-synuclein that were injected with (**a**) cerebellar extract from multiple system atrophy (MSA-P) case 1, (**b**) substantia nigra extract from Parkinson’s disease (PD) case 3 and (**c**) assembled recombinant human α-synuclein. (**d**) Spinal cord from a non-injected mouse was used as control. Scale bars, 50 μm

**Fig. 5:**
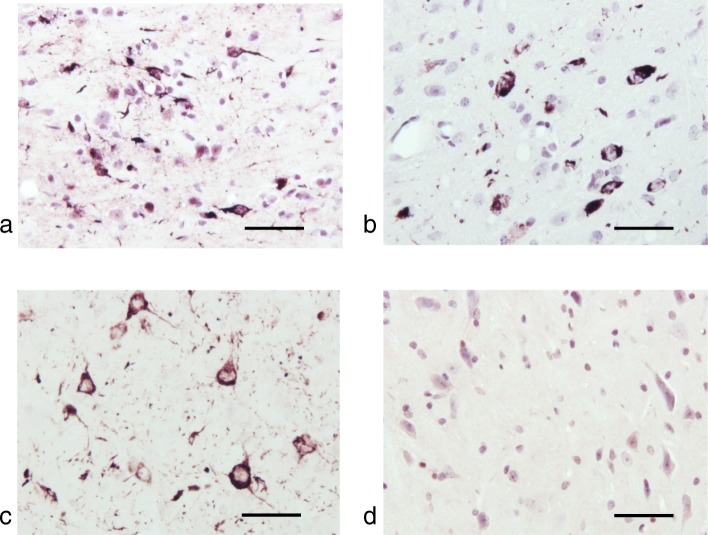
Immunohistochemistry for pS129-α-synuclein of midbrain from heterozygous mice transgenic for human mutant A53T α-synuclein that were injected with (**a**) cerebellar extract from multiple system atrophy (MSA-P) case 1, (**b**) substantia nigra extract from Parkinson’s disease (PD) case 3 and (**c**) assembled recombinant human α-synuclein. (**d**) Midbrain from a non-injected mouse was used as control. Scale bars, 50 μm

**Fig. 6:**
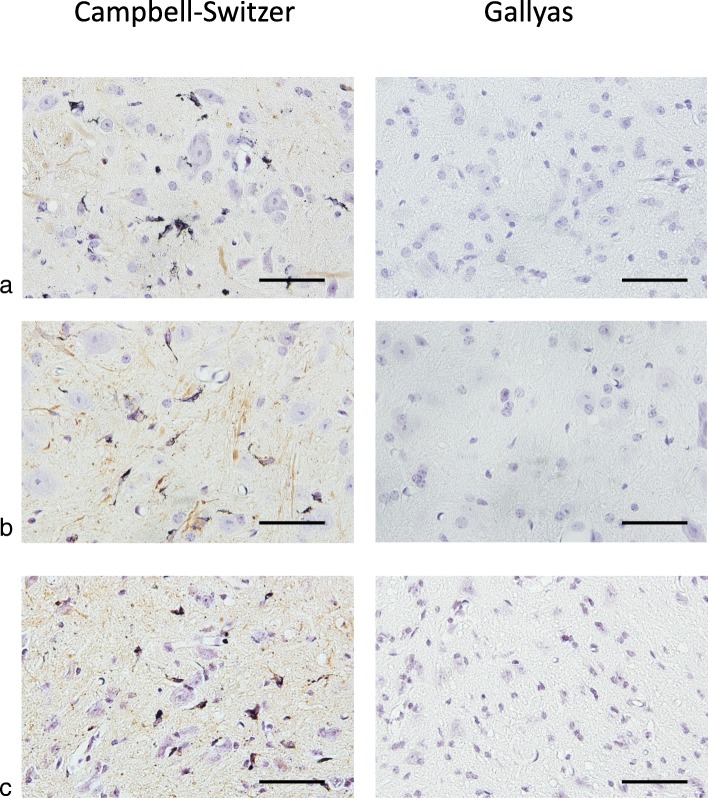
Silver staining (Campbell-Switzer and Gallyas) of lumbar spinal cord from heterozygous mice transgenic for human mutant A53T α-synuclein that were injected with (**a**) cerebellar extract from multiple system atrophy (MSA-P) case 1, (**b**) substantia nigra extract from Parkinson’s disease (PD) case 3 and (**c**) assembled recombinant human α-synuclein. Scale bars, 50 μm

Intracerebral injection of brain extracts from all cases of MSA and most cases of PD, as well as injection of assembled recombinant human α-synuclein, into heterozygous M83 mice resulted in staining for pS129-α-synuclein and motor dysfunction, leading to hindlimb paralysis (Figs. 1, 4 and 5). α-Synuclein inclusions were stained with Campbell-Switzer silver, but not Gallyas silver, irrespective of the injected material (Table 4; Fig. 6).

**Table 4:** Silver staining of sections from the central nervous system of heterozygous mice transgenic for human mutant A53T α-synuclein (tgM83^+/−^) following intracerebral injection of cerebellar homogenates from cases of multiple system atrophy (MSA) and substantia nigra homogenates from cases of Parkinson’s disease (PD). Sagittal brain sections encompassed brainstem, hippocampus and cerebral cortex. Spinal cord sections were of the lumbar region

Mouse	Human brain extract	Gallyas silver	Campbell-Switzer silver
tgM83 +/+	–	–	+
tgM83 +/−	–	–	–
tgM83 +/−	(1) MSA-P	–	+
tgM83 +/−	(2) MSA-P	–	+
tgM83 +/−	(3) MSA-P	–	+
tgM83 +/−	(4) MSA-P	–	+
tgM83 +/−	(5) MSA-P	–	+
tgM83 +/−	(6) MSA-C	–	+
tgM83 +/−	(7) MSA-C	–	+
tgM83 +/−	(1) PD	–	+
tgM83 +/−	(2) PD	–	+
tgM83 +/−	(3) PD	–	+
tgM83 +/−	(4) PD	–	+
tgM83 +/−	(5) PD	–	+

## Discussion

Intracerebral injection of cerebellar homogenates from 7 cases of MSA into heterozygous mice transgenic for human mutant A53T α-synuclein caused the formation of abundant neuronal α-synuclein inclusions and severe motor dysfunction. Most cases of MSA can be divided into MSA-P, a parkinsonian variant, and MSA-C, a cerebellar variant, based on the predominant motor symptoms [17, 29]. We injected brain extracts from 5 cases of MSA-P and 2 cases of MSA-C and did not observe any differences between cases. Future studies will have to look at additional cases of MSA-P and MSA-C. The time between injection and death was 167 ± 17 days. These findings confirm previous studies of MSA brain extract injections into the same mouse line [40, 52].

Unlike the earlier studies, which failed to observe a motor phenotype following intracerebral injection of PD homogenates [40], we observed neuronal α-synuclein inclusions and hindlimb paralysis in 7 of 17 mice injected with substantia nigra extracts from 5 PD patients. The time between injection and death was 286 ± 62 days. Five additional mice showed some α-synuclein inclusions, but no motor impairment at 18 months of age. Five mice failed to develop either inclusions or motor dysfunction. These results indicate that α-synuclein assembly preceded neurodegeneration. They are in agreement with previous studies showing that α-synuclein inclusions from PD and DLB brains exhibit prion-like behaviour [28, 41].

MSA homogenates were more potent than PD homogenates, consistent with the view that α-synuclein assemblies from MSA and PD are made of different conformers [38, 40, 52]. Negative stain electron microscopy established differences between α-synuclein filaments of DLB, MSA and PD [7, 43, 44, 46]. Previous work using recombinant α-synuclein showed that it can assemble into distinct filament conformations that exert differing effects [3, 37]. The structures of recombinant human α-synuclein assembled under various conditions also showed differences [20, 25, 48]. We do not know how the structures of our recombinant α-synuclein assemblies related to those reported by others [20, 25]. Structures of α-synuclein filaments from human brain are not known. It therefore remains to be seen how they relate to those of assembled recombinant protein. The structures of 3R tau filaments from Pick’s disease are different from those formed by incubating recombinant 2N3R tau with heparin [10, 54].

We quantified the amount of assembled α-synuclein in brain homogenates using dot blotting and an antibody specific for pS129-α-synuclein. Even though it has been reported that GCI-α-synuclein is less phosphorylated at S129 than Lewy body α-synuclein [38], the varying amounts of phosphorylated protein were unlikely to account for the differences in motor dysfunction that we observed between MSA and PD. Injection of as little as 1 ng assembled pS129-reactive α-synuclein was sufficient to cause motor dysfunction in heterozygous M83 mice 5 months later. However, this value may have been an underestimate, since some seed-competent species of assembled α-synuclein may not be phosphorylated at S129 [14].

Although the filamentous inclusions of PD and MSA are made of assembled α-synuclein, they can be distinguished by silver staining. Both types of inclusion stain with Campbell-Switzer, but only GCIs are also Gallyas-positive. This difference may reflect the presence of distinct conformers of assembled α-synuclein. It is reminiscent of tau filaments from Alzheimer’s disease, which are Campbell-Switzer and Gallyas-positive and those from Pick’s disease, which are only Campbell-Switzer-positive [50]. Electron cryo-microscopy has shown that these filaments are made of different conformers of assembled tau [10, 12].

Upon intracerebral injection of MSA and PD brain homogenates into heterozygous mice transgenic for human mutant A53T α-synuclein, inclusions were Campbell-Switzer-positive, but Gallyas-negative, like those in homozygous mice. A recent study has also shown that the inclusions formed following intracerebral injection of MSA brain extracts were Gallyas-negative [9]. Injection of assembled recombinant human α-synuclein gave rise to Campbell-Switzer-positive, Gallyas-negative α-synuclein inclusions. Cerebellar extract from a neurologically normal individual was without effect. These findings differ from those obtained following the injection of brain extract from APP/PS1 into APP23 transgenic mice, when the seeds determined the properties of the seeded aggregates [22].

α-Synuclein seeds from MSA and PD brains were from end-stage disease. The mechanisms resulting in seed formation at the beginning of the pathological process are unknown. This is particularly relevant for MSA, which is defined by the presence of abundant GCIs, despite the fact that α-synuclein is expressed at best at only low levels in oligodendrocytes and that assembly is concentration-dependent [2, 30]. α-Synuclein may give rise to a seed in as little as a single oligodendrocyte. Seeded aggregation could then proceed, even though oligodendrocytes express only low levels of α-synuclein. Assemblies have been shown to spread between oligodendrocytes [38]. The reason why GCIs were not observed following intracerebral injection of MSA brain extracts may have been due to the lack of significant transgene expression in oligodendrocytes. It remains to be seen if the A to T mutation at residue 53 of human α-synuclein also played a role. It will be interesting to determine the silver staining properties of the neuronal and oligodendroglial α-synuclein inclusions that have been described in cases of PD and DLB caused by mutations in *SNCA* [21, 24, 27, 36].

In conclusion, the present findings show that the silver staining properties of assembled α-synuclein in nerve cells following intracerebral injection of PD and MSA brain homogenates depend on both transgene expression and its cellular environment.
